# The genome of the basal agaricomycete *Xanthophyllomyces dendrorhous* provides insights into the organization of its acetyl-CoA derived pathways and the evolution of Agaricomycotina

**DOI:** 10.1186/s12864-015-1380-0

**Published:** 2015-03-25

**Authors:** Rahul Sharma, Sören Gassel, Sabine Steiger, Xiaojuan Xia, Robert Bauer, Gerhard Sandmann, Marco Thines

**Affiliations:** Biodiversity and Climate Research Centre (BiK-F), Georg-Voigt-Str. 14-16, 60325 Frankfurt (Main), Germany; Institute of Ecology, Evolution and Diversity, Goethe University, Max-von-Laue-Str. 9, 60323 Frankfurt (Main), Germany; Senckenberg Gesellschaft für Naturforschung, Senckenberganlage 25, 60325 Frankfurt (Main), Germany; Center for Integrative Fungal Research (IPF), Georg-Voigt-Str. 14-16, 60325 Frankfurt (Main), Germany; Department of Molecular Bioscience, J.W. Goethe University, Max-von-Laue-Str. 9, 60323 Frankfurt (Main), Germany; Institute of Evolution and Ecology, University of Tübingen, Auf der Morgenstelle 28, 72076 Tübingen, Germany

**Keywords:** Agaricomycotina, Astaxanthin Synthase, Fatty acid metabolism, Fungal evolution, *Xanthophyllomyces dendrorhous*, Phylogeny

## Abstract

**Background:**

*Xanthophyllomyces dendrorhous* is a basal agaricomycete with uncertain taxonomic placement, known for its unique ability to produce astaxanthin, a carotenoid with antioxidant properties. It was the aim of this study to elucidate the organization of its CoA-derived pathways and to use the genomic information of *X. dendrorhous* for a phylogenomic investigation of the Basidiomycota.

**Results:**

The genome assembly of a haploid strain of *Xanthophyllomyces dendrorhous* revealed a genome of 19.50 Megabases with 6385 protein coding genes. Phylogenetic analyses were conducted including 48 fungal genomes. These revealed Ustilaginomycotina and Agaricomycotina as sister groups. In the latter a well-supported sister-group relationship of two major orders, Polyporales and Russulales, was inferred. *Wallemia* occupies a basal position within the Agaricomycotina and *X. dendrorhous* represents the basal lineage of the Tremellomycetes, highlighting that the typical tremelloid parenthesomes have either convergently evolved in *Wallemia* and the Tremellomycetes, or were lost in the Cystofilobasidiales lineage. A detailed characterization of the CoA-related pathways was done and all genes for fatty acid, sterol and carotenoid synthesis have been assigned.

**Conclusions:**

The current study ascertains that *Wallemia* with tremelloid parenthesomes is the most basal agaricomycotinous lineage and that Cystofilobasidiales without tremelloid parenthesomes are deeply rooted within Tremellomycetes, suggesting that parenthesomes at septal pores might be the core synapomorphy for the Agaricomycotina. Apart from evolutionary insights the genome sequence of *X. dendrorhous* will facilitate genetic pathway engineering for optimized astaxanthin or oxidative alcohol production.

**Electronic supplementary material:**

The online version of this article (doi:10.1186/s12864-015-1380-0) contains supplementary material, which is available to authorized users.

## Background

*Xanthophyllomyces dendrorhous* (formerly often referred to as *Phaffia rhodozyma*) is a red-pigmented moderately psychrophilic growing yeast [1]. It is a basidiomycete classified among the Tremellomycetes in the order Cystofilobasidiales together with *Cystofilobasidium*, *Xanthophyllomyces*, and a clade containing *Mrakia*/*Mrakiella* and several anamorphic species of *Tausonia*/*Guehomyces*, *Itersonilia*, and *Udeniomyces* [2,3]. However, it is currently unclear whether the Cystifilobasidiales are the most basal group in the Tremellomycetes, or whether Cystofilobasidiales should be excluded from Tremellomycetes in order to assure its monophyly [2]. Because the Cytofilobasidiales are deeply rooted within the Agaricomycotina, they may be of key importance for understanding the evolution of this group. *Xanthophyllomyces dendrorhous* was originally isolated from exudates of *Betula* species and other broad-leave trees [1]. Later it was also isolated from leaves of *Nothofagus* trees and stromata of the tree’s biotrophic fungal parasite *Cyttaria* spp. [4]. *Xanthophyllomyces dendrorhous* possesses a homothallic life cycle [5]. A sexual reproductive cycle can be initiated by application of sugar alcohols [6] leading to sexual conjugation between cells of the same strain from which a long holobasidium with terminal spores is formed. Most publications show that *X. dendrorhous* is diploid [5,7]. However, electrophoretic chromosome separation in another strain [8] indicate that at least some strains may be haploid.

*Xanthophyllomyces dendrorhous* has two evolutionary special metabolic features. One is the synthesis of astaxanthin which is considered unique among fungi. The other is the fermentation of sugar to alcohol under oxidative conditions [9]. Astaxanthin serves as an antioxidant, quenching reactive oxygen species to protect *X. dendrorhous* from damage by oxidative stress [10,11]. Its biosynthesis is via the mevalonate pathway to the formation of β-carotene with enzymes similar to other carotenogenic fungi [12]. However, all steps of 3-hydroxylation and 4-ketolation at both terminal β-ionone rings leading to the formation of astaxanthin are carried out by a very unique P450 monooxygenase. This protein, Asy, belongs to the 3A subfamily [13]; electrons are provided by a specific cytochrome P450 reductase [14]. Although the astaxanthin concentration in wild-type strains of *X. dendrorhous* is too low for commercialization, attempts have been made to increase the astaxanthin yield developing *X. dendrorhous* as a production system for this carotenoid. The most promising yields were obtained by a combination of classical random mutagenesis followed by systematic engineering of the whole biosynthesis pathway [15].

Given the interesting position within the largest group of Basidiomycetes, the Agaricomycotina, its special metabolic features mentioned above related to acetyl-CoA derived pathways and its biotechnological potential, it was the aim of this study to elucidate the genome sequence for a phylogenomic investigation for the Agaricomycotina and to elucidate its acetyl-CoA metabolism. The latter is important to obtain tools for the analysis and the modeling of these biotechnological import pathways.

## Results

### Genome assemblies, completeness assessment and repeat elements

**Figure 1:**
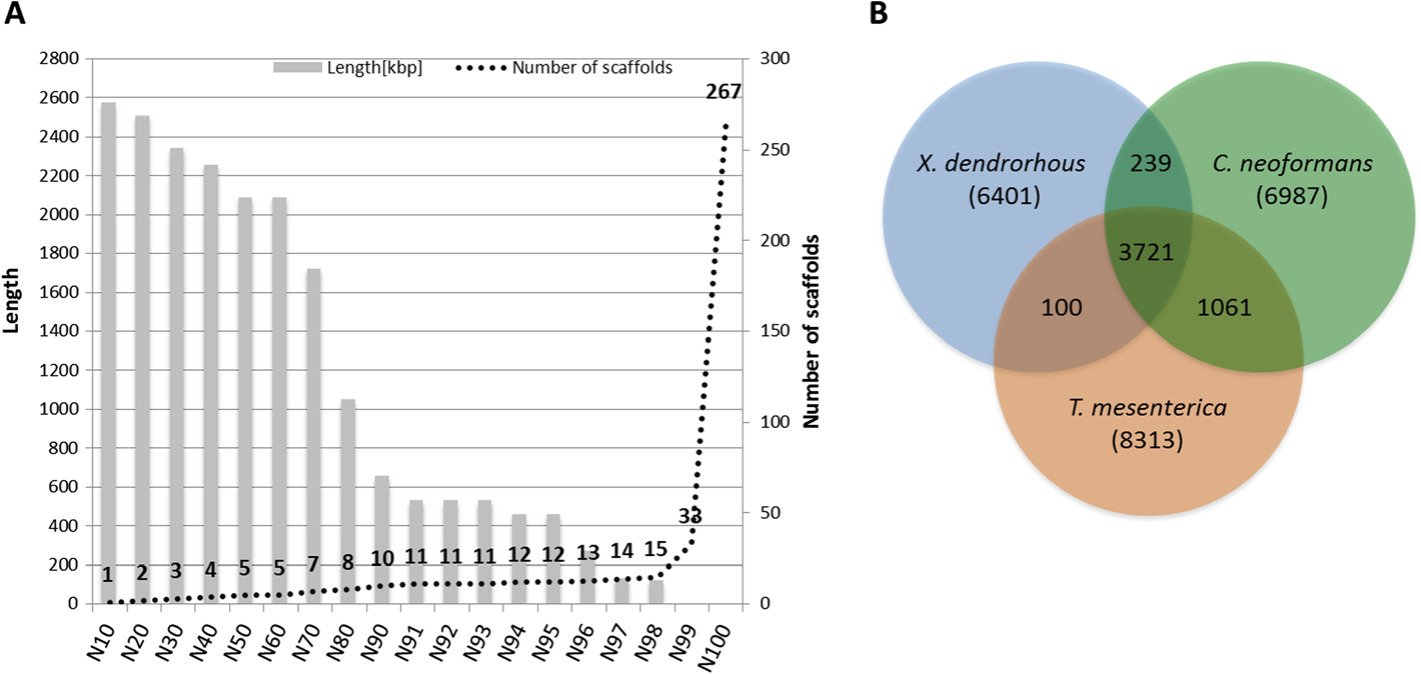
**Genome assembly quality plot and orthologs among three Tremellomycete genomes. (A)** Genome quality was assessed by defining N-classes for the assembled genome. Each N-class represents the N% of genome covered after sorting the assembled scaffolds from largest to smallest. The length of each N-class represents the length of shortest scaffold and the number of scaffolds represents the number of scaffolds in that particular N-class. For example N50 represents 50% of the genome covered in 5 scaffolds and the length of the N50^th^ scaffold is 2.08 Mb. **(B)** Venn diagram representing the orthologs shared by *Xanthophyllomyces dendrorhous, Cryptococcus neoformans* and *Tremella mesenterica*. Numbers in brackets represent the total number of protein coding genes predicted in these genomes.

Three Illumina libraries of insert size 250 bp [EMBL: ERR575093], 3 kb [EMBL: ERR575094] and 8 kb [EMBL: ERR575095], were sequenced on an Illumina HiSeq machine with 100 bp paired-end chemistry to generate the whole genome sequence of *X. dendrorhous*. After filtering raw reads using 26 phred score as average read quality cutoff and a 100 bp length cutoff, 93.23%, 20.13% and 24.46% of reads were left in the 250 bp, 3 kb and 8 kb insert libraries, respectively. Genome assemblies were done with the Velvet genome assembler [16] using the three different libraries with various k-mer lengths. The genome assembly resulted in a total of 267 scaffolds [EMBL: LN483084-LN483350], the nuclear genome was of 19.50 Mb in size in 257 scaffolds and the mitochondrial genome of 23.50 kb in 10 scaffolds. More than 70% of the genome was assembled into just 7 scaffolds, all of which were longer than 1.7 Mb, and 98% of the genome was represented by 15 scaffolds (Figure 1A).

After generating the genome assembly, all three libraries were mapped to the assembled genome, and around 96.03%, 92.61%, and 93.89% of the used reads could be mapped back to the assembled scaffolds. The genome completeness and continuity of the assembled genome were assessed by using the Cegma pipeline. Around 98.8% of the 458 core eukaryotic were recovered, indicating the high quality of the genome assembly. Repeat element predictions revealed 3.12% of repeat elements in the genome of *X. dendrorhous*. Classification of repeat elements was done by using TransposonPSI, and as the most abundant ones, 18 gypsy and 49 TY1_Copia retrotransposons were predicted in the assembled genome.

### Protein encoding genes and annotations

Both *ab-initio* and transcript alignment-based methods were used to generate the gene models for *X. dendrorhous*. These predictions generated 6385 protein coding genes. Functional annotations of the predicted proteome using Panther and InterPro revealed 4627 (72%) and 4951 (77.34%) protein sequences, respectively, which could be assigned with a function.

### Protein subcellular localization

The subcellular localization of *X. dendrorhous* proteins was predicted using ProtComp9 (http://linux1.softberry.com/) and 1378 proteins with mitochondrial, 68 with peroxisomal, 1789 with nuclear, 1420 with cytoplasmic and 705 with plasma membrane localisation were predicted. Secretome predictions using SignalP4.1, TargetP, and TmHmm resulted in a set of 296 proteins predicted to be secreted.

### CoA-related pathways

**Figure 2:**
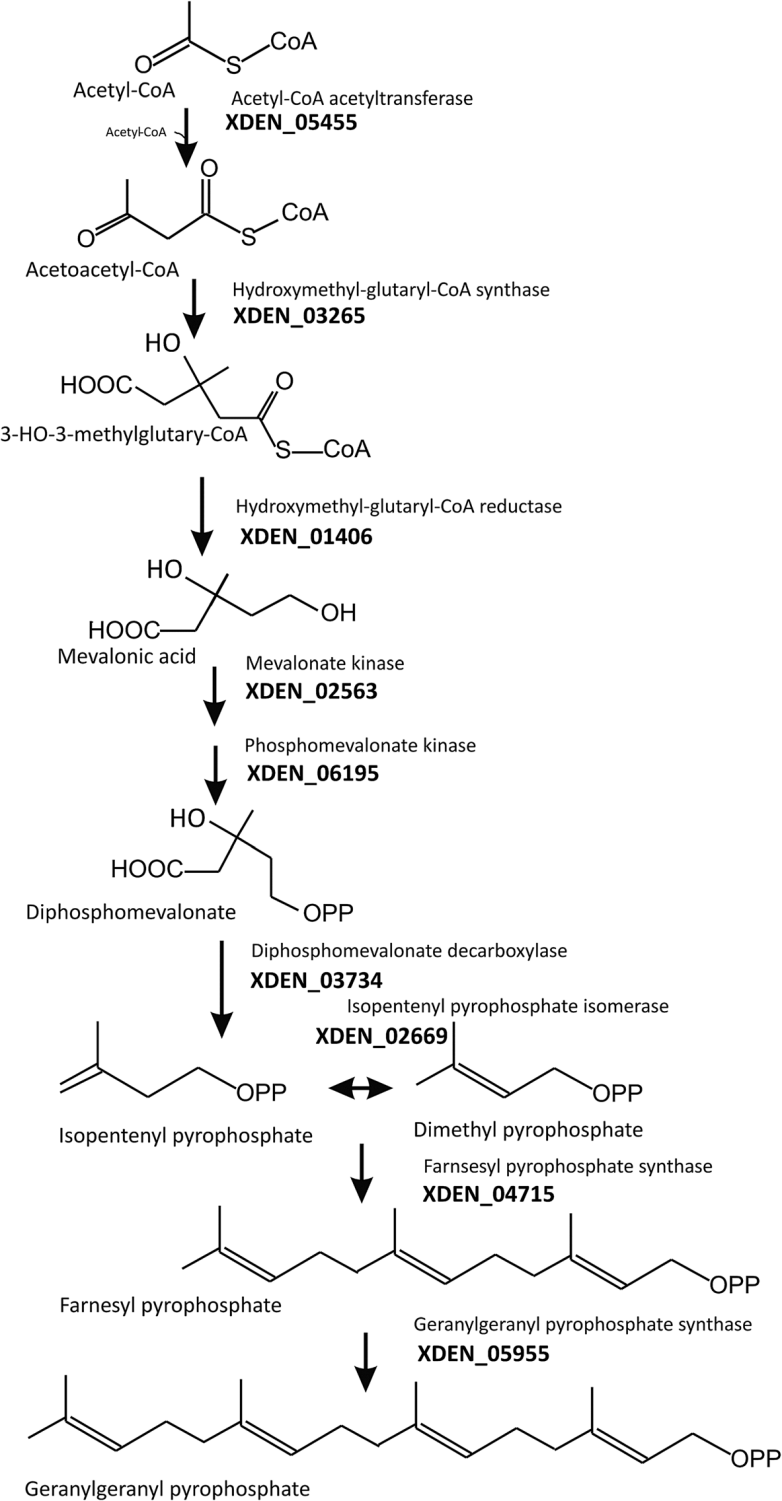
**The mevalonate pathway and synthesis of prenyl pyrophosphates.** Gene numbers from the *Xanthophyllomyces dendrorhous* genome are indicated next to the enzyme name, more details are shown in Table 1.

The two major acetyl-Co A derived biosynthesis pathways in *X. dendrorhous* are terpenoid and fatty acid biosynthesis. Typically, terpenoids in fungi are synthesized via the mevalonate pathway [17]. As outlined in Figure 2, this pathway starts with the condensation of three molecules of acetyl-CoA and proceeds via mevalonate and its diphosphorylation to isopentenyl pyrophosphate. The reactions of this pathway are catalyzed by six enzymes. All of these could be identified in the *X. dendrorhous* genome by comparison to the corresponding genes from related fungi (Table 1). The gene numbers are given next to the corresponding reactions in Figure 2. An alternative non-fungal route to isopentenyl pyrophosphate is via deoxyxylulose 5-phosphate [17]. All genes of this pathway are absent from the *X. dendrorhous* genome.

**Table 1:** **Genes of the mevalonate pathway and formation of prenyl pyrophosphates in** ***Xanthophyllomyces dendrorhous***

**Gene**	**Function**	**Gene number**	**Identity %**
ERG10	Acetyl-CoA acyltransferase	XDEN_05455	65% Cr.n.; 62% Rh.t.
ERG13	Hydroxymethylglutaryl-CoA synthase	XDEN_03265	59% Cr.n.; 57% Rh.t.
HMG1	Hydroxymethylglutaryl-CoA reductase	XDEN_01406	73% Cr.n.; 70% Rh.t.
ERG12	mevalonate kinase	XDEN_02563	36% C.g.; 52% Cr.n.
ERG8	phosphomevalonate kinase	XDEN_06195	41% Rh.t.; 36% Cr.n.
MVDD	Diphosphomevalonate decarboxylase	XDEN_03734	67% Cr.n.; 61% Rh.t.
IDi	Isopentenyl pyrophosphate isomerase	XDEN_02669	71% Cr.n.; 61% Rh.t.
*ERG* 20	Farnesyl pyrophosphate synthase	XDEN_03884	54% Cr.n.; 38% Rh.t.
crtE	Geranylgeranyl pyrophosphate synthase	XDEN_05955	49% Cr.n.; 45% Rh.t.

Rh.t. *Rhodosporidium toruloides.*

Cr.n. *Cryptococcus neoformans.*

**Figure 3:**
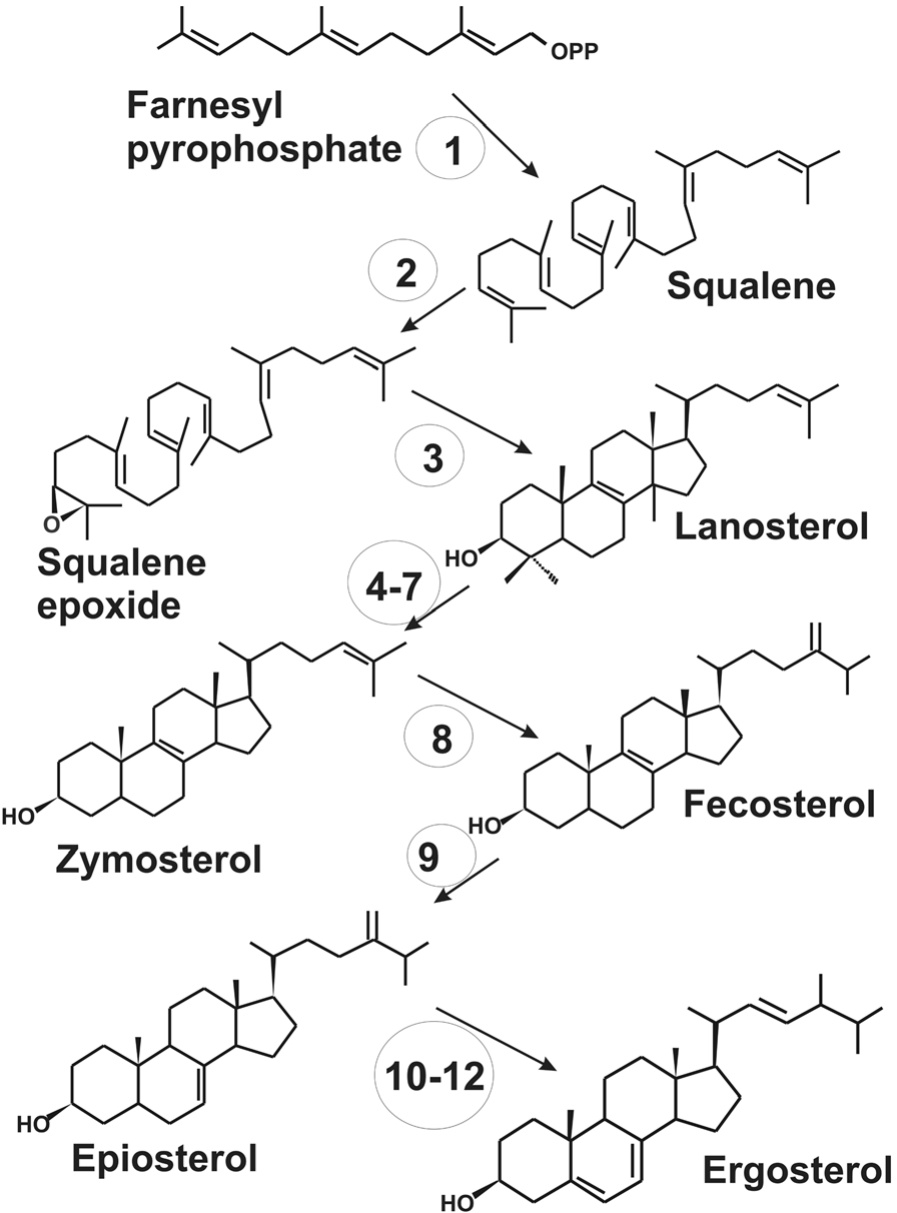
**The sterol biosynthesis pathway to ergosterol, the major sterol in** ***Xanthophyllomyces dendrorhous*** **.** Numbers indicate the individual genes/enzymes. Names and gene numbers from the *Xanthophyllomyces dendrorhous* genome are listed in Table 2.

After isomerization of isopentenyl pyrophosphate to dimethyl allyl pyrophosphate, the prenyl pyrophosphate chain is extended by condensation of isopentenyl pyrophosphate molecules with an allylic partner (Figure 2). In addition to the gene encoding isopentenyl pyrophosphate isomerase, two different prenyl transferases genes were detected encoding the enzymes for the formation of either farnesyl pyrophosphate the direct precursor of sterols or geranylgeranyl pyrophosphate the direct precursor of carotenoids (Table 1). The genes encoding isopentenyl pyrophosphate isomerase [18], geranylgeranyl pyrophosphate synthase [19] and farnesyl pyrophosphate synthase [20] have been cloned before from *X. dendrorhous*. Recently, it has been shown that these prenyl transferases act sequentially [20] as indicated in Figure 2. Ergosterol is the dominating sterol especially in higher fungi [21]. Its biosynthesis pathways is established in Figure 3 corresponding to the identified *ERG* genes. The genes assigned and listed in Table 2 encode the enzymes of the early steps including squalene synthesis, epoxidation and cyclisation to lanosterol and the genes involved in modification of this sterol. The reaction sequence involves a C-14 demethylase, a C-14 reductase, a C-3 dehydrogenase and a C-3 keto reductase yielding zymosterol. Next conversions are by C-24 methyl transferase to fecosterol by a C-8 isomerase to episterol and by a C-22 desaturase and a C-24 reductase to the final pathway product ergosterol. Among all genes of this sterol pathway, *ERG* 5 is the only gene cloned before from *X. dendrorhous* [22].

**Table 2:** **Genes of the sterol biosynthesis pathway leading to ergosterol in** ***Xanthophyllomyces dendrorhous***

Numbering*	**Enzyme**	**Gene**	**Gene in Phaffia genome**	Comparison to other fungal genes**
1	Squalene synthase	*ERG9*	DEN_03884	58% Rh t., 54% C.n.
2	Squalene epoxidase	*ERG1*	XDEN_03392	48% Cr.n., 42% M.c.
3	Lanosterol synthase	*ERG7*	XDEN_03169	58% Cr.n., 53% Rh.t.
4	Lanosterol demethylase	*ERG11*	XDEN_04688	63% Cr.n. , 50% Rh.t.
5	C-14 reductase	*ERG24*	XDEN_01380	55% Cr.n., 50% Rh.t.
6	C-3 dehydrogenase	*ERG26*	XDEN_04885	57% Cr.n., 50% Rh.t.
7	C-3 keto reductase	*ERG27*	XDEN_03960	31% Cr.n., 27% C.g.
8	C-24 methyl transferase	*ERG6*	XDEN_00954	67% Cr.n., 59% Rh.t.
9	C-8 isomerase	*ERG2*	XDEN_05343	66% Rh.t., 43% Cr.n.
10	C-5 desaturase	*ERG3*	XDEN_02355	55% Cr.n., 51% Rh.t.
11	C-3 desaturase	*ERG5*	XDEN_01040	64% Cr.n., 55% Rh.t.
12	C-24 reductase	*ERG4*	XDEN_03446	58% Cr.n., 44% Rh.t.

*number of enzyme refers to reactions in Figure 3 (sterol pathway);

**identity to Rh.t. *Rhodosporidium toruloides*, Cr.n. *Cryptococcus neoformans*, M.c. *Mucor circinelloides*, C.g. *Candida glabrata.*

All genes of carotenoid biosynthesis were known before our genome sequencing. Their numbers from the *X. dendrorhous* genome sequencing are XDEN_03692 for *crtYB* encoding the phytoene synthase/lycopene cyclase gene [23], XDEN_03755 for *crtI* the gene of a phytoene desaturase [24] and XDEN_04454 for the astaxanthin synthase gene [13]. XDEN_00679 is the gene coding for a reductase which provides the electrons for the P450-type astaxanthin synthase [14].

**Figure 4:**
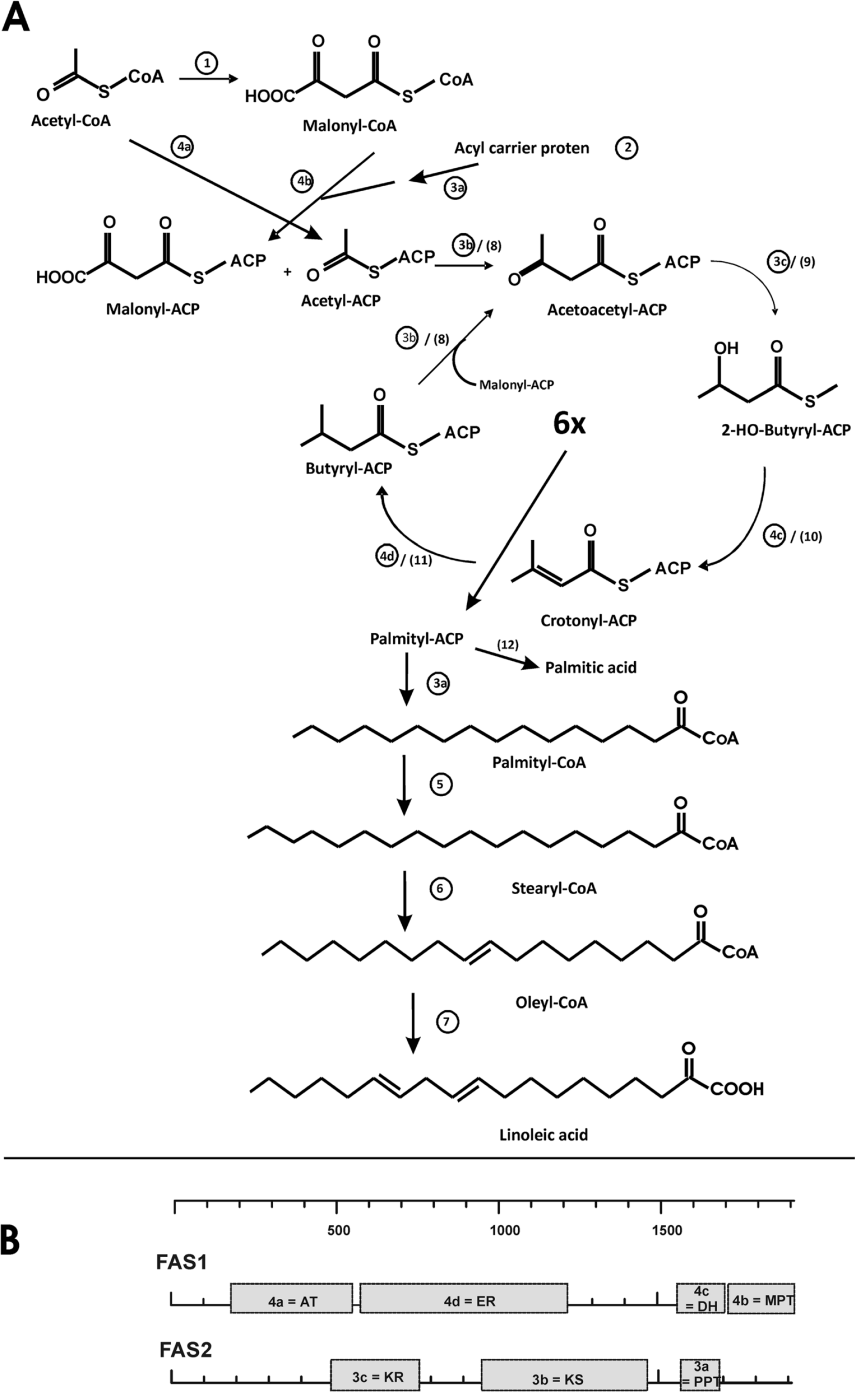
**Fatty acid metabolism. A**. Biosynthesis of cytoplasmic fatty acids (palmitic, oleic and linoleic acid) in *Xanthophyllomyces dendrorhous*. Numbers in circles indicate the individual enzymes and letters the individual domains on the fatty acid synthase (FAS) 1 and 2 complexes. Additional numbers in parenthesis refer to mitochondrial fatty acid synthesis. Names and gene numbers from the *X. dendrorhous* genome are listed in Table 3. **B**. Functional domains on the fatty acid synthase complex FAS1 and FAS2. AT malonyl transferase, ER enoyl reductase, DH hydroxyacyl dehydratase, MPT malonyl/palmitoyl transferase, KR ketoacyl reductase, KS ketoacyl synthase, PPT phosphopantetheinyl transferase. For details on these genes see Table 3.

In fungi, multiple fatty acid synthesis options are present which is structurally differently organized [25]. In the cytoplasm of eukaryotes, fatty acid synthesis operates with a multi-enzyme fatty acid synthase (FAS) comples (type I) with discrete functional domains for the individual reactions organized on two polypeptides. In addition, an independent mitochondrial (prokaryotic) synthesis pathway exists in fungi which uses independent enzymes (type II) encoded by separate genes [26]. In *X. dendrorhous*, the dominating fatty acids are palmitic, oleic and linoleic acid (unpublished results). Figure 4A shows the biosynthesis pathway to these fatty acids catalyzed by type I FAS. The synthesis starts with the acetyl CoA carboxylase and the acyl carrier protein (Table 3). The following reactions, the formation of acetyl-ACP and malonyl-ACP, condensations, ketoacyl reduction, the dehydratase reaction, and enoyl reduction all the way to palmityl-CoA are catalyzed two multi-enzyme complexes FAS1 and FAS2. The sequences of the individual domains could be identified and located on both FAS genes (Figure 4B). The additional genes involved in the elongation of C16 to C18 fatty acid and the insertion of a delta-9 and a delta-12 double bond were also identified in the *X. dendrorhous* genome. For the latter desaturase, we found two candidate genes.

**Table 3:** **Genes of cytoplasmic (top) and mitochondrial (bottom) fatty acid biosynthesis to linoleic acid in** ***Xanthophyllomyces dendrorhous***

Numbering*	**Gene name or KEGG EC**	**Enzyme**	**Gene ids** ***X. dendrorhous***	Comparison to other fungal genes**
1	ACC1	Acetyl CoA carboxylase	XDEN_00697	60% Cr.n.; 61% Rh.t.
2	ACP1	Acyl carrier protein	XDEN_03956	64% Cr. n.; 57% Rh. t.
3	FAS2	Fatty acid synthase complex subunit alpha	XDEN_04041	67% Cr.n.; 65% Rh.t.
4	FAS1	Fatty acid synthase complex subunit beta	XDEN_04566	64% Cr.n.; 50% M.c.
5	Elo1	Fatty acid elongase	XDEN_05837	61% Rh.t.; 60% Cr.n.
6	D9DES	Delta 9 fatty acid desaturase	XDEN_04179	68% Cr.n. ; 59% Rh.t.
7	D12DES	Delta 12 fatty acid desaturase	XDEN_05333	59% Cr.n.; 54% Rh.t.
			XDEN_00895	54% Cr.n.; 48% Rh.t.
8	MCT1	Acetyl CoA acyltransferase 2	XDEN_05455	63% Cr.n.; 62% Rh.t.
9	OAR1	ketoacyl reductase	XDEN_04398	42% C.n.; 43% S.c.
10	MFE2p	Enoyl-CoA hydratase	XDEN_02337	58% Rh.t.; 54% Cr.n.
11	1.1.1.35	3-hydroxyacyl CoA dehydrogenase	XDEN_01855	72% Cr.n.; 71% Rh.t.
12	3.1.2.22	Palmitoyl thioesterase	XDEN_03361	42% Cr.n.

*number of enzyme refers to reactions in Figure 4 (fatty acid synthesis);

**identity to Rh.t. *Rhodosporidium toruloides*, Cr.n. *Cryptococcus neoformans*, M.c. *Mucor circinelloides*, C.g. *Candida glabrata*, S.c. *Saccharomyces cerevisiae*.

Enzymes/Genes numbering 1 to 7 from the cytoplasmic pathway, numbers 8 to 9 from the mitochondrial pathway.

### Secondary metabolism analyses

Genes involving in the secondary metabolism apart from terpenoids were predicted by using the SMURF [27] online web server and secondary metabolite clusters were defined. Only one polyketide synthase (PKS) like and two non-ribosomal peptide synthetases (NRPS) like genes were predicted from the genome of *X. dendrorhous*. Two candidate secondary metabolite clusters were predicted (Additional file 1: Table S1). Backbone genes of these clusters have been listed in the Additional file 1: Table S2. Further InterPro domain analyses of the PKS-like gene predicted a beta-ketoacyl synthase (KS) domain, an acyl transferase (AT) domain and an acyl carrier protein (ACP) domain within XDEN_04041*.*

### Orthology analyses among Tremellales

*Xanthophyllomyces dendrorhous* protein sequences were tested for orthology with the two other available Tremellomycetes genomes, i.e. *Cryptococcus neoformans* (teleomorph *Filobasidiella neoformans*) and *Tremella mesenterica*. A total of 3721 orthologs are shared by all of the three genomes, 239 orthologs are shared by only *X. dendrorhous* and *C. neoformans*, while *C. neoformans* and *T. mesenterica* (Tremellales) share 1061 orthologs not present in *X. dendrorhous* (Cystofilobasidials) (Figure 1B), highlighting that *X. dendrorhous* is not closely related to *C. neoformans*, supporting the splitting of the Tremellomycetes into the Cystofilobasidiales on the one hand and the core Tremellomycetes (Filobasidiales, Holtermanniales, Trichosporonales, Tremellales) on the other.

### Phylogenetic analyses

**Figure 5:**
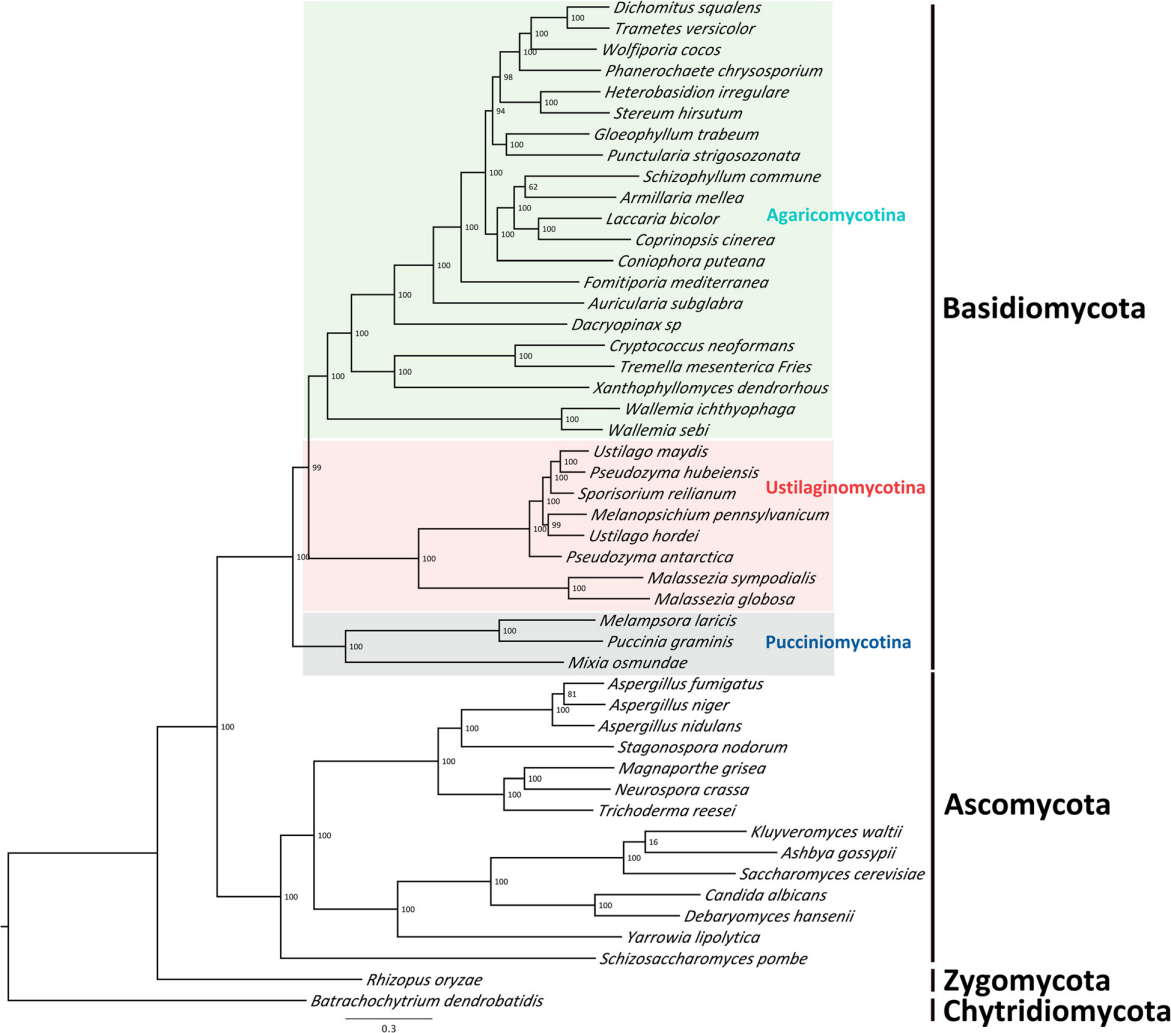
**Phylogenetic analyses considering 48 fungal genomes.** Phylogenetic analysis was conducted by predicting orthologs within all 48 genomes fungal genomes using orthoMCL. Ortholog predictions generated 137 1:1 orthologs in all 48 genomes. Multiple sequence alignments were performed and a maximum likelihood tree was generated using RAxML. Numbers on branches denote support from 1000 bootstrap replicates.

Phylogenetic analyses were done using 48 fungal genomes (Additional file 1: Table S3), including the genome of *X. dendrorhous*. Orthologs among these genomes were identified, and a total of 636 orthologs were predicted in all these 48 genomes. Of these, 137 were 1:1 orthologs, which were used to perform the phylogenetic analyses. The maximum likelihood tree generated using RAxML was supported by high to maximum support for all nodes and revealed a sister-group relationship of Agaricomycotina and Ustilaginomycotina (Figure 5). *Wallemia* was placed basal within the Agaricomycotina and *X. dendrorhous* appeared as sister taxon to *C. neoformans* and *T. mesenterica*. In general, the Tremellomycetes with *Xanthophyllomyces*, but without *Wallemia*, were revealed as monophyletic and to be the sister clade to the remaining Agaricomycotina. For the Agaricomycotina, the Dacryomycetes were confirmed as the sister-group to the Agaricomycetes. Within the Agaricomycetes, the Auriculales occupied the basal position, followed by the Hymenochetales, which were revealed as the sister-group of the other Agaricomycetes included with maximum support. Boletales and Agaricales were found to group together with maximum support and formed the sister group to a clade comprising the orders Corticiales, Gloeophyllales, Russulales, and Polyporales. Within this group, which was supported by a 94% bootstrap support, the former as well as the latter two orders were grouped together with maximum and high support, respectively. This is also in line with the results of the Bayesian phylogenetic inference (Additional file 2: Figure S2).

## Discussion

### Genome assembly and completeness

Over the past few years next generation sequencing technologies have extensively been used to elucidate the whole genome and transcriptome of fungal species [28-32]. In this work Illumina sequencing have been used for sequencing the genome and transcriptome of a red-pigmented yeast, *Xanthophyllomyces dendrorhous*. The of the fact that more than 70% of the genome were represented by only 7 scaffolds of more than 1.7 Mb in size and that 98% of the genome was covered by just 15 scaffolds highlights that the genome has been assembled almost to chromosome-length scaffolds. The high quality of the genome assembly is also suggested by a recovery of about 98.8% of the core eukaryotic genes in a CEGMA analysis. This is most likely the result of using an appropriate combination of short paired-read as well as long distance mate-pair libraries and the fact that the strain we sequenced appears to be haploid. The low content of repeat elements of only about 3.1% further facilitates the genome assembly.

Secondary metabolite encoding genes have been described in several fungal genomes [33-37]. The *X. dendrorhous* genome encodes for only one polyketide synthase like gene and two non-ribosomal peptide synthetase like genes. This suggests a limited ability to produce biologically active substances as expected for species not depending on keeping other organisms at bay.

### Agaricomycete phylogeny

In line with earlier phylogenomic studies using fewer taxa [38,39] a sister-group relationship of Agaricomycotina and Ustilaginomycotina was inferred. With respect to septal pore ultrastructure it was interesting to note that with the inclusion of the cystofilobasidiomycete *X. dendrorhous*, *Wallemia* spp. remained in the most basal position within the Agaricomycotina. The septal pore apparatus of *Wallemia* with the typical tremelloid sacculate parenthesomes closely resembles that of the core Tremellomycetes (Filobasidiales, Holtermanniales, Trichosporonales, Tremellales), whereas the dolipores of Cystofilobasidiales lack parenthesomes [3,39]. Thus either the typical tremelloid sacculate parenthesomes at the septal pores were lost in the Cystofilobasidiales lineage, or have convergently evolved in *Wallemia* and the core Tremellomycetes, Assuming that it is unlikely that the complex tremelloid parenthosomes have evolved twice, a loss of the tremelloid parenthosomes in the Cytofilobasidiales seems to be the more parsimonious explanation. In this sense the evolution of the Agaricomycotina was apparently accompanied by the evolutionary development of parenthesomes at the septal pores that may improve the cell to cell communication [40].

In addition, our study shows with optimal support the monophyly of the group consisting of Tremellomycetes and Cystofilobasidiales. Based on morphological, ultra-structural, chemical, ecological and molecular data, the monophyly of the Tremellomycetes (incl. the Cystofilobasidiales) has been suggested by various authors [3]. However, this had not been previously supported by molecular phylogenetic studies [41-43].

Within the Agaricomycetes, the phragmobasidial Auriculales were in a basal position and the Hymenochaetales with holobasidia were placed basal to the crown-group of the class. In contrast to an earlier phylogenomic study [44], phylogenetic relationships within the crown of Agaricomycetes showed a high resolution. The recently-described orders Gloeophyllales and Corticiales [45] were clustered together with maximum support, in line with the overview provided by Hibbett [46], and placed basal to a clade containing the Agaricomycetidae (represented by Agaricales and Boletales) and a second clade that included Polyporales and Russulales. The sister-group relationship of Polyporales and Russulales was supported by a bootstrap support of 98% in the phylogenomic analysis and is in contrast to the result from a multi-locus dataset [46], which discussed the order Russulales as a sister group to the Agaricomycetidae. The same topology was obtained using Bayesian Inference, with high support (Additional file 2: Figure S2).

### CoA-related metabolic pathways and beyond

The two prominent terpenoid pathways in *X. dendrorhous* lead to the synthesis of sterols and carotenoids. They are of biotechnological importance due to the ongoing development of this fungus as a biological astaxanthin production system [15]. In addition, it offers the potential for the hetrologous synthesis of novel sesquiterpenes like α–cuprene [47] or diterpenes instead of carotenoids. Annotation of all genes of terpenoid synthesis in *X. dendrorhous* starting with the mevalonate pathway and ending with ergosterol and astaxanthin was successful (Tables 1 and 2). Among these were the genes of two different prenyl transferases which sequentially provide C15 farnesyl pyrophosphate for sterol and geranylgeranyl pyrophosphate for carotenoid biosynthesis [20]. *Xanthophyllomyces dendrorhous* possesses a unique astaxanthin synthase related the cytochrome P450 3A subfamily with an unknown phylogenetic origin [13]. The highest similaritiy to fungal cytochrome P450 oxidases was found in *Cryptococcus neoformans,* but with only 36% identity.

The genes for specific biosynthesis pathways are often clustered in fungal genomes. This is not the case for sterol and carotenoid biosynthesis in the genome of *X. dendrorhous* and is in contrast to carotenogenic fungi from other groups in which these genes are organized in clusters. All carotenogenic fungi possess the *crtYB* and *crtI* genes. For example in *Phycomyces blakesleeanus* and *Mucor circinelloides*, both genes are found next to each other with a spacing of 1.4 or 0.5 kbp, respectively, but convergently transcribed [48] and in *Fusarium fujikuroi*, they are 0.6 kbp apart and transcribed together in the same direction [49].

Another important group of compounds originating from acetyl-CoA are the fatty acids. In the *X. dendrorhous* genome, the genes for the cytoplasmic pathway and the mitochondrial pathway could be discriminated (Table 3). The latter operates on individual enzymes [26] for which all genes could be annotated. The gene organization of the cytoplasmic pathway is more complex. In ascomycetes, the genes for two fatty acid synthase proteins 1 and 2 exist with all necessary eight enzymatic activities (Figure 4A). In contrast, most basidiomycetes like *Laccaria*, *Coprinopsis* and *Ustilago* possess a single very large protein with all necessary fatty acid synthesis activities [50]. However, this is not the case in *X. dendrorhous*. Here, we found the genes for two distinct fatty acid synthase proteins 1 and 2 (Figure 4B) which resembles the situation in the related species *Cryptococcus neoformans*. However, we were unable to identify the subunit of the acyl-carrier protein, neither on FAS2 as in yeast nor on FAS1 as in *C. neoformans* [50].

Even under aerobic conditions, *X. dendrorhous* grows fermentative on glucose accumulating ethanol, which, at the beginning of the stationary phase, is re-used as growth substrate [51]. Since carotenoid biosynthesis is highest in the oxidative phase, it is important to understand the unknown regulatory mechanisms responsible for optimum astaxanthin synthesis on different substrates. The whole genome sequence of *X. dendrorhous* now provides a source to address the genes of the primary metabolism, providing a basis for transcriptomic and metabolomic analysis. This should be helpful to look for regulatory circuits and metabolic networks which supply acetyl-CoA as substrate. The current study also provides genomic data from a species of the Agaricomycotina for setting up a basis for the comparison with other fungi to investigate how the C30 and C40 terpenoid pathways have developed.

## Conclusions

The current study provides the first insights into a genome of a cystofilobasidiomycete and reveals that *Wallemia* is the most basal agaricomycotinous lineage, followed by the Tremellomycetes with a sister-group relationship between the Cystofilobasidiales and the core Tremellomycetes. Thus, this study provides further insights into the evolution of Agaricomycotina and suggests that the typical cisternal caps (parenthesomes) at the septal pores represent an apomorphic characteristic for the Argaricomycotina in general. Accordingly, the lack of parenthesomes at the septal pores may be apomorphic only for the Cystofilobasidiales. Phylogenomic investigations also support a sister-group relationship of Agaricomycotina and Ustilaginomycotina. Within the Agaricomycotina, the phylogenetic relationships of the species included were resolved with high to maximum support and provided evidence for a sister-group relationship of Polyporales and Russulales. With respect to the biotechnological potential of *X. dendrorhous*, the genome sequence will extremely facilitate genetic pathway engineering of secondary products. All genes of acetyl-Co A derived pathways could be annotated. They can be used to overproduce existing fatty acids and sterols in addition to carotenoids or extend these pathways yielding new products. Furthermore, the accessibility of genes of the primary metabolisms is extremely helpful to model and engineer an optimum precursor supply.

## Methods

### Growth and isolation of genomic DNA

The *X. dendrorhous* strain CBS6938 (= ATCC96594) was grown as shaking culture in YPD medium at 21°C for 5 days. The pellet from 15 ml of culture was suspended in 0.5 ml YPD and mixed with 300 μl of glass beads (0.25 mm-0.5 mm diameter). The cells were broken in a swing mill (Retsch MM200) at a frequency of 30/s. After centrifugation, the supernatant was collected and purified by extraction with phenol/chloroform/isoamylalcohol. Finally, the DNA was precipitated by adding 2.5 volumes of 100% ice-cold ethanol and 1/10 volume of a 3 M sodium acetate solution overnight at −20°C. The DNA was pelleted, washed with 70% ice-cold ethanol and dried at room temperature. The DNA pellet was suspended in 30 μl H_2_O and stored at 4°C. The amount of isolated DNA was determined from an agarose gel after staining with ethidium bromide by densitometry of the fluorescence and comparison to standard DNA of known amounts.

### Isolation of RNA

RNA was extracted by using NucleoSpin® RNA Plant kit (MACHEREY-NAGEL GmbH & Co. KG) according to the instructions of the manufacturer. The sample cultivation conditions for the RNA isolation are the same as above. The RNA quality was controlled using a NanoPhotometer (IMPLEN) as well as being evaluated on a 1.5% agarose gel stained with ethidium bromide.

### Preprocessing of genomic and transcriptomic reads

Data filtering parameter estimations and data filtering steps were performed on adapter/primer trimmed data using FastQFS (Sharma and Thines, unpublished). A length cutoff of 100 bps and an average quality cutoff of 26 phred score was used to filter reads from all three libraries. RNA-Seq data was filtered using Trimmomatic [52], with a length cutoff of 32 and a quality cutoff of 15 in a window of 5 bp.

### Genome assembly, genome assembly completeness assessment and repeat element masking

The genome of *X. dendrorhous* was assembled using the Velvet [16] genome assembler. All three libraries of insert sizes of 250 bps, 3 kb, and 8 kb were used to generate scaffolds. Velvet was optimized by testing several k-mer sizes and k-mer coverage cutoffs. An optimal assembly was generated by a k-mer of a length of 93 and using a k-mer coverage cutoff of 15. The completeness of the genome assembly was assessed using the CEGMA [53] pipeline. Repeat elements within the assembled genome were predicted using RepeatModeler (http://www.repeatmasker.org/RepeatModeler.html). Both Recon [54] and RepeatScout [55] tools were used within the RepeatModeler pipeline for *de novo* repeat element predictions. Reference-based repeat element search was done using the Repbase libraries v20130422 [56]. Tandem repeat elements were identified using trf [57] within the RepeatModeler pipeline and the final set of predicted repeat elements were masked by using RepeatMasker (http://www.repeatmasker.org/). Repeat element characterizations were also done by using TransposonPSI (http://transposonpsi.sourceforge.net/).

### Gene prediction and annotation

Both *ab initio* and alignment-based methods were used to predict protein coding genes within the assembled genome. Genemark-ES [58] was used for generating the first set of gene models. These gene models were tested for RNA-seq coverage greater than 10X using Samtools [59]. The gene models supported by RNA-Seq were further used for training Augustus [60]. RNA-seq reads were mapped on the assembled genome by using Tophat2 [61] and transcripts were generated by using Cufflinks [62]. The resulting bam file from Tophat2 was used to generate Intron/exon hints from Augustus predictions.

The transcript sequences obtained from Cufflinks were mapped on the genome by using PASA [63] and GMAP [64]. These gene models and the information from GeneMark-ES, Augustus, PASA and GMAP was used for obtaining consensus gene models by using EVM [65]. High weights were given to the transcript-mapped gene models. In another round of gene predictions, RNA-Seq data was again mapped to the gene masked and repeat masked genome. Newly obtained transcripts were added to the gene models generated by the first round of gene predictions (Additional file 1: Figure S1).

Gene annotations were performed using Blast2GO [66]. Protein family analyses were done using the standalone versions of PANTHER [67]. KEGG [68] analyses were performed using the KAAS [69] online server. The euKaryotic Orthologous Group cluster (KOG) [70] analyses was performed locally by downloading KOG protein sequences; and alignments were done using the standalone BlastP [71] with an e-value cutoff of e-5. Protein domain analysis was done using Interproscan [72]. TribeMCL [73] was used for the clustering of protein sequences. For the annotation of the biosynthesis pathways, biochemical information of the enzymes were searched in the KEGG database (http://www.genome.jp/kegg/kegg2.html), the BRENDA Enzyme Information system (http://www.brenda-enzymes.de/index.php4?page=information/introduction.php4) and at NCBI.

### Protein subcellular localization

Protein subcellular localization was predicted using ProtComp9 (http://linux1.softberry.com/). Proteins having an extracellular secretion signal were predicted using SignalP v4.1 [74]. These outputs were further filtered using TargetP v1 [75] and TmHmm [76] predictions, for excluding proteins targeted to the mitochondrion or containing transmembrane domains, respectively.

### Orthology and phylogenetic analyses

In total 48 fungal genomes were used for the identification of orthologous genes for conducting a phylogenomic analysis. Ortholog predictions were done considering all protein sequences of 48 fungal genomes using OrthoMCL [77]. OrthoMCL was run using a percentage identity cutoff of 50% and an e-value cutoff of e^−5^. Multiple sequence alignments of 1:1 orthologs were performed using Mafft [78] with the G-INS-i algorithm. Maximum Likelihood phylogenetic inference on the concatenated set was done using RAxML [79], using the GAMAWAG model and 1000 bootstrap replicates. In another approach MrBayes [80] was run on the aligned protein sequences using 2 million generations, sampling every 500th tree and discarding the first 95% of the trees sampled before inferring posterior probability values. For reference of the specific parameters, the Bayes block has been deposited at http://dx.doi.org/10.12761/SGN.2015.1.

### Data access

All 3 genomic sequence libraries [EMBL: ERR575093-ERR575095] and a RNA-Seq library [EMBL: ERR575096] have been submitted to the European Nucleotide Archive (ENA) database (Study accession number: PRJEB6925). The assembled scaffolds of *X. dendrorhous* and annotations have also been submitted to ENA and can be accessed from accession ids LN483084-LN483350. Genome and annotation files are also available at our local server and can be accessed from http://dx.doi.org/10.12761/SGN.2015.1.

## Additional files

Additional file 1: Table S1. Secondary metabolite clusters predicted within the genome of *X. dendrorhous.* **Table S2.** Backbone genes of the two secondary metabolite clusters predicted within the genome of *X. dendrorhous*. **Table S3.** List of fungal genomes used for phylogenetic analyses. **Figure S1.** Gene prediction pipeline used for predicting genes within the genome of *X. dendrorhous*. Additional file 2: Figure S2. Phylogenetic tree based on Bayesian phylogenetic inference. Numbers on branches denote posterior probabilities.

## References

[1] M. W. Miller MY, Masami S (1976). Phaffia, a new yeast genus in the deuteromycotina (Blastomycetes). Int J Syst Evol Microbiol.

[2] John Webster RW (2007). Introduction to fungi.

[3] Weiß M BR, Sampaio JP, Oberwinkler F (2014). Tremellomycetes and related groups.

[4] David-Palma M, Libkind D, Sampaio JP (2014). Global distribution, diversity hot spots and niche transitions of an astaxanthin-producing eukaryotic microbe. Mol Ecol.

[5] Kucsera J, Pfeiffer I, Ferenczy L (1998). Homothallic life cycle in the diploid red yeast Xanthophyllomyces dendrorhous (Phaffia rhodozyma). Antonie Van Leeuwenhoek.

[6] Golubev WI (1995). Perfect state of Rhodomyces dendrorhous (Phaffia rhodozyma). Yeast.

[7] Hermosilla G, Martinez C, Retamales P, Leon R, Cifuentes V (2003). Genetic determination of ploidy level in Xanthophyllomyces dendrorhous. Antonie Van Leeuwenhoek.

[8] Wery J, Gutker D, Renniers AC, Verdoes JC, van Ooyen AJ (1997). High copy number integration into the ribosomal DNA of the yeast Phaffia rhodozyma. Gene.

[9] Reynders MB, Rawlings DE, Harrison STL (1997). Demonstration of the Crabtree effect in Phaffia rhodozyma during continuous and fed-batch cultivation. Biotechnol Lett.

[10] William A, Schroeder EAJ (1993). Antioxidant role of carotenoids in Phaffia rhodozyma. Microbiology.

[11] William A, Schroeder EAJ (1995). Carotenoids protectPhaffia rhodozyma against singlet oxygen damage. J Ind Microbiol.

[12] Sandmann G MN, Karl Esser PAL, Bennett JW, Heinz D (2002). The mycota X industrial applications. The Mycota X industrial applications.

[13] Ojima K, Breitenbach J, Visser H, Setoguchi Y, Tabata K, Hoshino T (2006). Cloning of the astaxanthin synthase gene from Xanthophyllomyces dendrorhous (Phaffia rhodozyma) and its assignment as a beta-carotene 3-hydroxylase/4-ketolase. Mol Genet Genomics.

[14] Alcaino J, Barahona S, Carmona M, Lozano C, Marcoleta A, Niklitschek M (2008). Cloning of the cytochrome p450 reductase (crtR) gene and its involvement in the astaxanthin biosynthesis of Xanthophyllomyces dendrorhous. BMC Microbiol.

[15] Gassel S, Breitenbach J, Sandmann G (2014). Genetic engineering of the complete carotenoid pathway towards enhanced astaxanthin formation in Xanthophyllomyces dendrorhous starting from a high-yield mutant. Appl Microbiol Biotechnol.

[16] Zerbino DR, Birney E (2008). Velvet: algorithms for de novo short read assembly using de Bruijn graphs. Genome Res.

[17] Eisenreich W, Rohdich F, Bacher A (2001). Deoxyxylulose phosphate pathway to terpenoids. Trends Plant Sci.

[18] Verdoesa JC, Ooyenab AJJ (1999). Isolation of the isopentenyl diphosphate isomerase encoding gene of Phaffia rhodozyma; improved carotenoid production in Escherichia coli. Acta Botanica Gallica: Botany Lett.

[19] Breitenbach J, Visser H, Verdoes JC, van Ooyen AJ, Sandmann G (2011). Engineering of geranylgeranyl pyrophosphate synthase levels and physiological conditions for enhanced carotenoid and astaxanthin synthesis in Xanthophyllomyces dendrorhous. Biotechnol Lett.

[20] Alcaino J, Romero I, Niklitschek M, Sepulveda D, Rojas MC, Baeza M (2014). Functional characterization of the Xanthophyllomyces dendrorhous farnesyl pyrophosphate synthase and geranylgeranyl pyrophosphate synthase encoding genes that are involved in the synthesis of isoprenoid precursors. PLoS One.

[21] Weete JD, Abril M, Blackwell M (2010). Phylogenetic distribution of fungal sterols. PLoS One.

[22] Loto I, Gutierrez MS, Barahona S, Sepulveda D, Martinez-Moya P, Baeza M (2012). Enhancement of carotenoid production by disrupting the C22-sterol desaturase gene (CYP61) in Xanthophyllomyces dendrorhous. BMC Microbiol.

[23] Verdoes JC, Krubasik KP, Sandmann G, van Ooyen AJ (1999). Isolation and functional characterisation of a novel type of carotenoid biosynthetic gene from Xanthophyllomyces dendrorhous. Mol Gen Genet.

[24] Verdoes JC, Misawa N, van Ooyen AJ (1999). Cloning and characterization of the astaxanthin biosynthetic gene encoding phytoene desaturase of Xanthophyllomyces dendrorhous. Biotechnol Bioeng.

[25] Schweizer E, Hofmann J (2004). Microbial type I fatty acid synthases (FAS): major players in a network of cellular FAS systems. Microbiol Mol Biol Rev.

[26] Schneider R, Brors B, Massow M, Weiss H (1997). Mitochondrial fatty acid synthesis: a relic of endosymbiontic origin and a specialized means for respiration. FEBS Lett.

[27] Khaldi N, Seifuddin FT, Turner G, Haft D, Nierman WC, Wolfe KH (2010). SMURF: genomic mapping of fungal secondary metabolite clusters. Fungal Genet Biol.

[28] Janbon G, Ormerod KL, Paulet D, Byrnes EJ, Yadav V, Chatterjee G (2014). Analysis of the genome and transcriptome of Cryptococcus neoformans var. grubii reveals complex RNA expression and microevolution leading to virulence attenuation. PLoS Genet.

[29] Toome M, Ohm RA, Riley RW, James TY, Lazarus KL, Henrissat B (2014). Genome sequencing provides insight into the reproductive biology, nutritional mode and ploidy of the fern pathogen Mixia osmundae. New Phytol.

[30] Morita T, Koike H, Hagiwara H, Ito E, Machida M, Sato S (2014). Genome and transcriptome analysis of the basidiomycetous yeast Pseudozyma antarctica producing extracellular glycolipids, mannosylerythritol lipids. PLoS One.

[31] Floudas D, Binder M, Riley R, Barry K, Blanchette RA, Henrissat B (2012). The Paleozoic origin of enzymatic lignin decomposition reconstructed from 31 fungal genomes. Science.

[32] Sharma R, Mishra B, Runge F, Thines M (2014). Gene loss rather than gene gain is associated with a host jump from monocots to dicots in the smut fungus Melanopsichium pennsylvanicum. Genome Biol Evol.

[33] Dean RA, Talbot NJ, Ebbole DJ, Farman ML, Mitchell TK, Orbach MJ (2005). The genome sequence of the rice blast fungus Magnaporthe grisea. Nature.

[34] Ehrlich KC, Yu J, Cotty PJ (2005). Aflatoxin biosynthesis gene clusters and flanking regions. J Appl Microbiol.

[35] Xu J, Saunders CW, Hu P, Grant RA, Boekhout T, Kuramae EE (2007). Dandruff-associated Malassezia genomes reveal convergent and divergent virulence traits shared with plant and human fungal pathogens. Proc Natl Acad Sci U S A.

[36] Yu J, Bhatnagar D, Cleveland TE (2004). Completed sequence of aflatoxin pathway gene cluster in Aspergillus parasiticus. FEBS Lett.

[37] Yu J, Chang PK, Ehrlich KC, Cary JW, Bhatnagar D, Cleveland TE (2004). Clustered pathway genes in aflatoxin biosynthesis. Appl Environ Microbiol.

[38] Zajc J, Liu Y, Dai W, Yang Z, Hu J, Gostincar C (2013). Genome and transcriptome sequencing of the halophilic fungus Wallemia ichthyophaga: haloadaptations present and absent. BMC Genomics.

[39] Padamsee M, Kumar TK, Riley R, Binder M, Boyd A, Calvo AM (2012). The genome of the xerotolerant mold Wallemia sebi reveals adaptations to osmotic stress and suggests cryptic sexual reproduction. Fungal Genet Biol.

[40] Bloemendal S, Kuck U (2013). Cell-to-cell communication in plants, animals, and fungi: a comparative review. Naturwissenschaften.

[41] Bauer RBD, Sampaio JP, Weiß M, Oberwinkler F (2006). The simple-septate basidiomycetes: a synopsis. Mycol Prog.

[42] Matheny PBGJ, Zalar P, Arun Kumar TK, Hibbett DS (2006). Resolving the phylogenetic position of the Wallemiomycetes: an enigmatic major lineage of Basidiomycota. Can J Bot.

[43] Millanes AM, Diederich P, Ekman S, Wedin M (2011). Phylogeny and character evolution in the jelly fungi (Tremellomycetes, Basidiomycota, Fungi). Mol Phylogenet Evol.

[44] Ebersberger I, de Matos SR, Kupczok A, Gube M, Kothe E, Voigt K (2012). A consistent phylogenetic backbone for the fungi. Mol Biol Evol.

[45] Hibbett DS, Binder M, Bischoff JF, Blackwell M, Cannon PF, Eriksson OE (2007). A higher-level phylogenetic classification of the Fungi. Mycol Res.

[46] Hibbett DS (2006). A phylogenetic overview of the Agaricomycotina. Mycologia.

[47] Melillo E, Setroikromo R, Quax WJ, Kayser O (2013). Production of alpha-cuprenene in Xanthophyllomyces dendrorhous: a step closer to a potent terpene biofactory. Microb Cell Fact.

[48] Sanz C, Velayos A, Alvarez MI, Benito EP, Eslava AP (2011). Functional analysis of the Phycomyces carRA gene encoding the enzymes phytoene synthase and lycopene cyclase. PLoS One.

[49] Linnemannstons P, Prado MM, Fernandez-Martin R, Tudzynski B, Avalos J (2002). A carotenoid biosynthesis gene cluster in Fusarium fujikuroi: the genes carB and carRA. Mol Genet Genomics.

[50] Reich M, Gobel C, Kohler A, Buee M, Martin F, Feussner I (2009). Fatty acid metabolism in the ectomycorrhizal fungus Laccaria bicolor. New Phytol.

[51] Wozniak A, Lozano C, Barahona S, Niklitschek M, Marcoleta A, Alcaino J (2011). Differential carotenoid production and gene expression in Xanthophyllomyces dendrorhous grown in a nonfermentable carbon source. FEMS Yeast Res.

[52] Bolger AM, Lohse M, Usadel B (2014). Trimmomatic: a flexible trimmer for Illumina sequence data. Bioinformatics.

[53] Parra G, Bradnam K, Korf I (2007). CEGMA: a pipeline to accurately annotate core genes in eukaryotic genomes. Bioinformatics.

[54] Bao Z, Eddy SR (2002). Automated de novo identification of repeat sequence families in sequenced genomes. Genome Res.

[55] Price AL, Jones NC, Pevzner PA (2005). De novo identification of repeat families in large genomes. Bioinformatics.

[56] Jurka J, Kapitonov VV, Pavlicek A, Klonowski P, Kohany O, Walichiewicz J (2005). Repbase update, a database of eukaryotic repetitive elements. Cytogenet Genome Res.

[57] Benson G (1999). Tandem repeats finder: a program to analyze DNA sequences. Nucleic Acids Res.

[58] Borodovsky M, Lomsadze A (2011). Eukaryotic gene prediction using GeneMark.hmm-E and GeneMark-ES. Curr Protoc Bioinformatics.

[59] Li H, Handsaker B, Wysoker A, Fennell T, Ruan J, Homer N (2009). The Sequence Alignment/Map format and SAMtools. Bioinformatics.

[60] Stanke M, Schoffmann O, Morgenstern B, Waack S (2006). Gene prediction in eukaryotes with a generalized hidden Markov model that uses hints from external sources. BMC Bioinformatics.

[61] Kim D, Pertea G, Trapnell C, Pimentel H, Kelley R, Salzberg SL (2013). TopHat2: accurate alignment of transcriptomes in the presence of insertions, deletions and gene fusions. Genome Biol.

[62] Trapnell C, Williams BA, Pertea G, Mortazavi A, Kwan G, van Baren MJ (2010). Transcript assembly and quantification by RNA-Seq reveals unannotated transcripts and isoform switching during cell differentiation. Nat Biotechnol.

[63] Haas BJ, Delcher AL, Mount SM, Wortman JR, Smith RK, Hannick LI (2003). Improving the Arabidopsis genome annotation using maximal transcript alignment assemblies. Nucleic Acids Res.

[64] Wu TD, Watanabe CK (2005). GMAP: a genomic mapping and alignment program for mRNA and EST sequences. Bioinformatics.

[65] Haas BJ, Salzberg SL, Zhu W, Pertea M, Allen JE, Orvis J (2008). Automated eukaryotic gene structure annotation using EVidenceModeler and the Program to Assemble Spliced Alignments. Genome Biol.

[66] Conesa A, Gotz S, Garcia-Gomez JM, Terol J, Talon M, Robles M (2005). Blast2GO: a universal tool for annotation, visualization and analysis in functional genomics research. Bioinformatics.

[67] Finn RD, Tate J, Mistry J, Coggill PC, Sammut SJ, Hotz HR (2008). The Pfam protein families database. Nucleic Acids Res.

[68] Kanehisa M, Goto S (2000). KEGG: kyoto encyclopedia of genes and genomes. Nucleic Acids Res.

[69] Moriya Y, Itoh M, Okuda S, Yoshizawa AC, Kanehisa M (2007). KAAS: an automatic genome annotation and pathway reconstruction server. Nucleic Acids Res.

[70] Koonin EV, Fedorova ND, Jackson JD, Jacobs AR, Krylov DM, Makarova KS (2004). A comprehensive evolutionary classification of proteins encoded in complete eukaryotic genomes. Genome Biol.

[71] Altschul SF, Gish W, Miller W, Myers EW, Lipman DJ (1990). Basic local alignment search tool. J Mol Biol.

[72] Quevillon E, Silventoinen V, Pillai S, Harte N, Mulder N, Apweiler R (2005). InterProScan: protein domains identifier. Nucleic Acids Res.

[73] Enright AJ, Van Dongen S, Ouzounis CA (2002). An efficient algorithm for large-scale detection of protein families. Nucleic Acids Res.

[74] Petersen TN, Brunak S, von Heijne G, Nielsen H (2011). SignalP 4.0: discriminating signal peptides from transmembrane regions. Nat Methods.

[75] Emanuelsson O, Nielsen H, Brunak S, von Heijne G (2000). Predicting subcellular localization of proteins based on their N-terminal amino acid sequence. J Mol Biol.

[76] Krogh A, Larsson B, von Heijne G, Sonnhammer EL (2001). Predicting transmembrane protein topology with a hidden Markov model: application to complete genomes. J Mol Biol.

[77] Li L, Stoeckert CJ, Roos DS (2003). OrthoMCL: identification of ortholog groups for eukaryotic genomes. Genome Res.

[78] Katoh K, Standley DM (2013). MAFFT multiple sequence alignment software version 7: improvements in performance and usability. Mol Biol Evol.

[79] Stamatakis A (2006). RAxML-VI-HPC: maximum likelihood-based phylogenetic analyses with thousands of taxa and mixed models. Bioinformatics.

[80] Ronquist F, Teslenko M, van der Mark P, Ayres DL, Darling A, Hohna S (2012). MrBayes 3.2: efficient Bayesian phylogenetic inference and model choice across a large model space. Syst Biol.

