# Supplementary material for: The genome of the basal agaricomycete Xanthophyllomyces dendrorhous provides insights into the organization of its acetyl-CoA derived pathways and the evolution of Agaricomycotina
Source: BMC Genomics. 2015 Mar 25;16(1):233. doi: 10.1186/s12864-015-1380-0 (PMC4393869; doi:10.1186/s12864-015-1380-0)
Supplement: Additional file 1: Table S1. — Secondary metabolite clusters predicted within the genome of X. dendrorhous. Table S2. Backbone genes of the two secondary metabolite clusters predicted within the genome of X. dendrorhous. Table S3. List of fungal genomes used for phylogenetic analyses. Figure S1. Gene prediction pipeline used for predicting genes within the genome of X. dendrorhous. [file 12864_2015_1380_MOESM1_ESM.pdf]

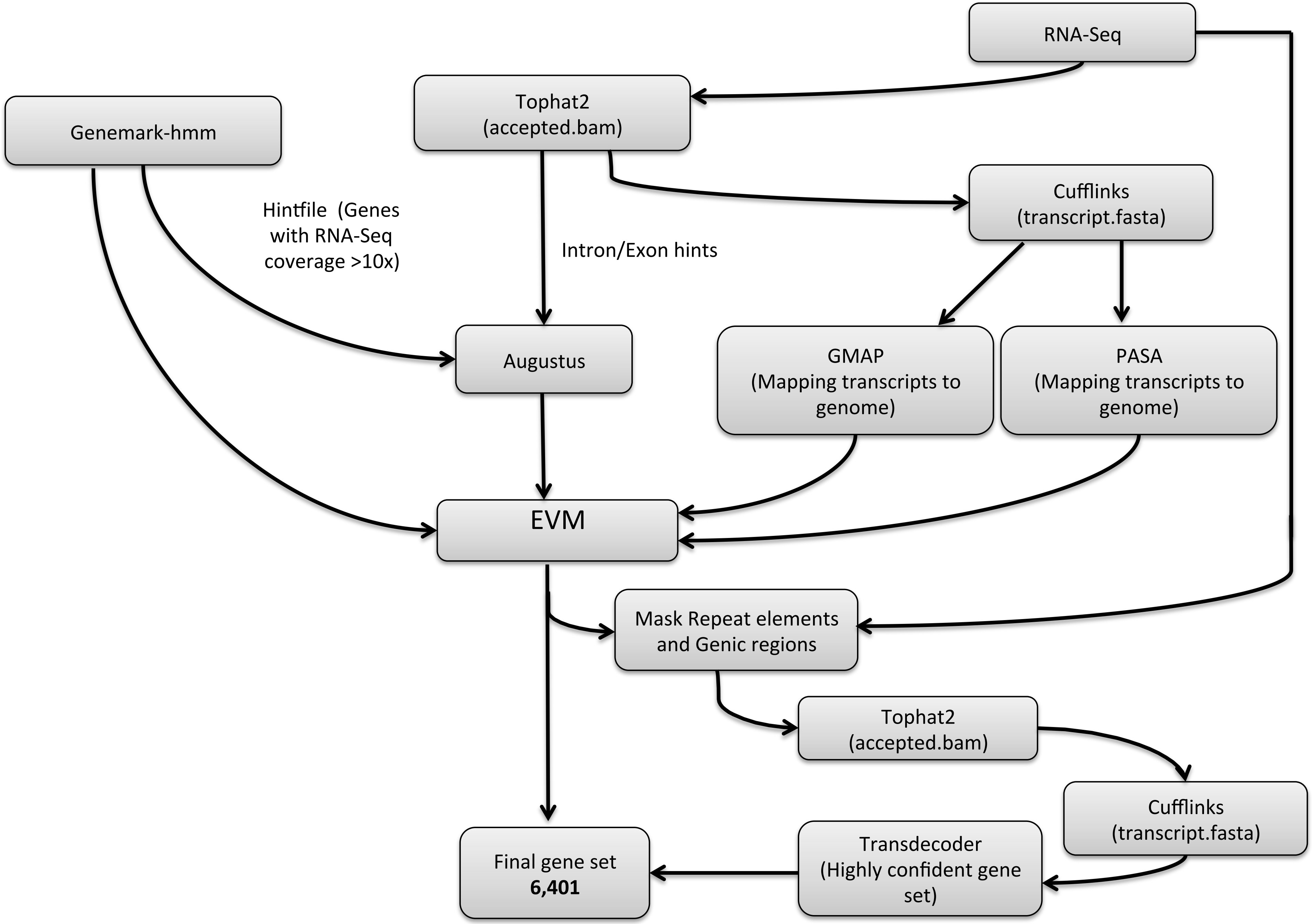

Supplementary Figure 1: Gene prediction pipeline used for predicting genes within the genome of *X. dendrorhous*.

Supplementary Table 1: Secondary metabolite clusters predicted within the genome of *X. dendrorhous* .

Cluster:1

**Backbone_gene_id**	**Gene_id**	**Gene_positions**	**Chromosome-Contig**	**Gene_order**	**5'end**	**3'end**	**Gene_distance**	**Domain_score**
XDEN_04041	XDEN_04042	-1	54	241	740866	744314	541	1
XDEN_04041	XDEN_04041	0	54	240	733506	740325	0	1
XDEN_04041	XDEN_04040	1	54	239	731589	733193	313	0
XDEN_04041	XDEN_04039	2	54	238	727989	730753	836	1
XDEN_04041	XDEN_04038	3	54	237	726536	727498	491	0
XDEN_04041	XDEN_04037	4	54	236	723179	726257	279	1
XDEN_04041	XDEN_04036	5	54	235	720173	722704	475	0
XDEN_04041	XDEN_04035	6	54	234	711884	719170	1003	1
XDEN_04041	XDEN_04034	7	54	233	706419	710916	968	0
XDEN_04041	XDEN_04033	8	54	232	704246	705780	639	0
XDEN_04041	XDEN_04032	9	54	231	702306	704043	203	1
XDEN_04041	XDEN_04031	10	54	230	701197	702001	305	0
XDEN_04041	XDEN_04030	11	54	229	699396	700978	219	0
XDEN_04041	XDEN_04029	12	54	228	697265	699076	320	1
XDEN_04041	XDEN_04028	13	54	227	692878	695960	1305	1
XDEN_04041	XDEN_06315	14	54	226	691217	692606	272	0
XDEN_04041	XDEN_04027	15	54	225	688706	691035	182	1
**Cluster:2**								
**Backbone_gene_id**	**Gene_id**	**Gene_positions**	**Chromosome-Contig**	**Gene_order**	**5'end**	**3'end**	**Gene_distance**	**Domain_score**
XDEN_06121	XDEN_06131	-10	79	695	2062902	2064575	357	0
XDEN_06121	XDEN_06130	-9	79	694	2061014	2062545	778	1
XDEN_06121	XDEN_06129	-8	79	693	2059803	2060236	501	0
XDEN_06121	XDEN_06128	-7	79	692	2057630	2059302	697	1
XDEN_06121	XDEN_06127	-6	79	691	2052204	2056933	859	0
XDEN_06121	XDEN_06126	-5	79	690	2050708	2051345	246	0
XDEN_06121	XDEN_06125	-4	79	689	2049048	2050462	418	0
XDEN_06121	XDEN_06124	-3	79	688	2047721	2048630	456	0

XDEN_06121	XDEN_06123	-2	79	687	2045652	2047265	469	0
XDEN_06121	XDEN_06122	-1	79	686	2044728	2045183	295	0
XDEN_06121	XDEN_06121	0	79	685	2040016	2044433	0	1
XDEN_06121	XDEN_06120	1	79	684	2038248	2039550	466	0
XDEN_06121	XDEN_06119	2	79	683	2035799	2037908	340	1
XDEN_06121	XDEN_06118	3	79	682	2032319	2033759	2040	0
XDEN_06121	XDEN_06117	4	79	681	2029048	2032016	303	1
XDEN_06121	XDEN_06116	5	79	680	2027516	2028720	328	1
XDEN_06121	XDEN_06115	6	79	679	2023522	2027484	32	1
XDEN_06121	XDEN_06114	7	79	678	2019987	2023027	495	0
XDEN_06121	XDEN_06113	8	79	677	2018817	2019767	220	0
XDEN_06121	XDEN_06112	9	79	676	2017106	2018316	501	0
XDEN_06121	XDEN_06111	10	79	675	2014526	2017015	91	0
XDEN_06121	XDEN_06110	11	79	674	2012525	2013825	701	0
XDEN_06121	XDEN_06109	12	79	673	2011746	2012307	218	0
XDEN_06121	XDEN_06108	13	79	672	2008849	2011495	251	1
XDEN_06121	XDEN_06107	14	79	671	2006362	2008318	531	0
XDEN_06121	XDEN_06106	15	79	670	2002723	2005517	845	1

Supplementary Table 2: Backbone genes of the two secondary metabolite clusters predicted within the genome of *X. dendrorhous* .

Backbone_gene_id	Scaffold id	Gene_order	5'_end	3'_end	SMURF_backbone_gene_prediction
XDEN_00460	162	343	1032809	1037153	NRPS-Like
XDEN_04041	54	240	733506	740325	PKS-Like
XDEN_06121	79	685	2040016	2044433	NRPS-Like

Supplementary Table 3: List of fungal genomes used for phylogenetic analyses.

	Species names	Taxonomy*	Reference
1	*Melampsora laricis*	Basidiomycota; Pucciniomycotina; Pucciniomycetes; Pucciniales;	[1]
2	*Puccinia graminis*	Basidiomycota; Pucciniomycotina; Pucciniomycetes; Pucciniales;	[1]
3	*Mixia osmundae*	Basidiomycota; Pucciniomycotina; Mixiomycetes; Mixiales;	[2]
4	*Malassezia sympodialis*	Basidiomycota; Ustilaginomycotina; Malasseziomycetes; Malasseziales	[3]
5	*Pseudozyma antarctica*	Basidiomycota; Ustilaginomycotina; Ustilaginomycetes; Ustilaginales;	[4]
6	*Pseudozyma hubeiensis*	Basidiomycota; Ustilaginomycotina; Ustilaginomycetes; Ustilaginales;	[5]
7	*Ustilago maydis*	Basidiomycota; Ustilaginomycotina; Ustilaginomycetes; Ustilaginales;	[6]
8	*Ustilago hordei*	Basidiomycota; Ustilaginomycotina; Ustilaginomycetes; Ustilaginales;	[7]
9	*Malassezia globosa*	Basidiomycota; Ustilaginomycotina; Malasseziomycetes; Malasseziales;	[8]
10	*Sporisorium reilianum*	Basidiomycota; Ustilaginomycotina; Ustilaginomycetes; Ustilaginales;	[9]
11	*Melanopsichium pennsylvanicum*	Basidiomycota; Ustilaginomycotina; Ustilaginomycetes; Ustilaginales;	[10]
12	*Auricularia subglabra*	Basidiomycota; Agaricomycotina; Agaricomycetes; Agaricomycetes incertae sedis; Auriculariales;	[11]
13	*Coniophora puteana*	Basidiomycota; Agaricomycotina; Agaricomycetes; Agaricomycetidae; Boletales;	[11]
14	*Dichomitus squalens*	Basidiomycota; Agaricomycotina; Agaricomycetes; Agaricomycetes incertae sedis; Polyporales;	[11]
15	*Fomitiporia mediterranea*	Basidiomycota; Agaricomycotina; Agaricomycetes; Agaricomycetes incertae sedis; Hymenochaetales;	[11]
16	*Gloeophyllum trabeum*	Basidiomycota; Agaricomycotina; Agaricomycetes; Agaricomycetes incertae sedis; Gloeophyllales;	[11]
17	*Punctularia strigosozonata*	Basidiomycota; Agaricomycotina; Agaricomycetes; Agaricomycetes incertae sedis; Corticiales;	[11]
18	*Stereum hirsutum*	Basidiomycota; Agaricomycotina; Agaricomycetes; Agaricomycetes incertae sedis; Russulales;	[11]
19	*Trametes versicolor*	Basidiomycota; Agaricomycotina; Agaricomycetes; Agaricomycetes incertae sedis; Polyporales;	[11]
20	*Tremella mesenterica*	Basidiomycota; Agaricomycotina; Tremellomycetes; Tremellales;	[11]
21	*Wolfiporia cocos*	Basidiomycota; Agaricomycotina; Agaricomycetes; Agaricomycetes incertae sedis;	[11]

		Polyporales;	
22	*Dacryopinax sp.*	Basidiomycota; Agaricomycotina; Dacrymycetes; Dacrymycetales;	[11]
23	*Cryptococcus neoformans*	Basidiomycota; Agaricomycotina; Tremellomycetes; Tremellales;	[12]
24	*Heterobasidion irregulare*	Basidiomycota; Agaricomycotina; Agaricomycetes; Agaricomycetes incertae sedis; Russulales;	[13]
25	*Laccaria bicolor*	Basidiomycota; Agaricomycotina; Agaricomycetes; Agaricomycetidae; Agaricales;	[14]
26	*Armillaria mellea*	Basidiomycota; Agaricomycotina; Agaricomycetes; Agaricomycetidae; Agaricales;	[15]
27	*Coprinopsis cinerea*	Basidiomycota; Agaricomycotina; Agaricomycetes; Agaricomycetidae; Agaricales;	[16]
28	*Phanerochaete chrysosporium*	Basidiomycota; Agaricomycotina; Agaricomycetes; Agaricomycetes incertae sedis; Polyporales;	[17]
29	*Schizophyllum commune*	Basidiomycota; Agaricomycotina; Agaricomycetes; Agaricomycetidae; Agaricales;	[18]
30	*Wallemia sebi*	Basidiomycota; Basidiomycota incertae sedis; Wallemiomycetes; Wallemiales;	[19]
31	*Wallemia ichthyophaga*	Basidiomycota; Basidiomycota incertae sedis; Wallemiomycetes; Wallemiales;	[20]
32	*Aspergillus fumigatus*	Ascomycota; saccharomyceta; Pezizomycotina; leotiomyceta; Eurotiomycetes; Eurotiomycetidae; Eurotiales;	[21]
33	*Saccharomyces cerevisiae*	Ascomycota; saccharomyceta; Saccharomycotina; Saccharomycetes; Saccharomycetales;	[22]
34	*Schizosaccharomyces pombe*	Ascomycota; Taphrinomycotina; Schizosaccharomycetes; Schizosaccharomycetales;	[23]
35	*Stagonospora nodorum*	Ascomycota; saccharomyceta; Pezizomycotina; leotiomyceta; dothideomyceta; Dothideomycetes; Pleosporomycetidae; Pleosporales;	[24]
36	*Trichoderma reesei*	Ascomycota; saccharomyceta; Pezizomycotina; leotiomyceta; sordariomyceta; Sordariomycetes; Hypocreomycetidae; Hypocreales;	[25]
37	*Yarrowia lipolytica*	Ascomycota; saccharomyceta; Saccharomycotina; Saccharomycetes; Saccharomycetales;	[26]
38	*Aspergillus nidulans*	Ascomycota; saccharomyceta; Pezizomycotina; leotiomyceta; Eurotiomycetes; Eurotiomycetidae; Eurotiales;	[27]
39	*Candida albicans*	Ascomycota; saccharomyceta; Saccharomycotina; Saccharomycetes; Saccharomycetales;	[28]
40	*Aspergillus niger*	Ascomycota; saccharomyceta; Pezizomycotina; leotiomyceta; Eurotiomycetes; Eurotiomycetidae; Eurotiales;	[29]
41	*Debaryomyces hansenii*	Ascomycota; saccharomyceta; Saccharomycotina; Saccharomycetes;	[26]

		Saccharomycetales;	
42	*Kluyveromyces waltii*	Ascomycota; saccharomyceta; Saccharomycotina; Saccharomycetes; Saccharomycetales;	[30]
43	*Ashbya gossypii*	Ascomycota; saccharomyceta; Saccharomycotina; Saccharomycetes; Saccharomycetales;	[31]
44	*Magnaporthe grisea*	Ascomycota; saccharomyceta; Pezizomycotina; leotiomyceta; sordariomyceta; Sordariomycetes; Sordariomycetidae; Magnaporthales;	[32]
45	*Neurospora crassa*	Ascomycota; saccharomyceta; Pezizomycotina; leotiomyceta; sordariomyceta; Sordariomycetes; Sordariomycetidae; Sordariales;	[33]
46	*Batrachochytrium dendrobatidis*	Chytridiomycota; Chytridiomycetes; Rhizophydiales;	Broad-Mit[a]
47	*Rhizopus oryzae*	Fungi incertae sedis; Early diverging fungal lineages; Mucoromycotina; Mucorales;	[34]

* Information extracted from NCBI Taxonomy Browser.
[a] Broad-FGI, MIT/Harvard Broad Institute, funded through the Fungal Genome Initiative;

References

1. Duplessis S, Cuomo CA, Lin YC, Aerts A, Tisserant E, Veneault-Fourrey C, Joly DL, Hacquard S, Amselem J, Cantarel BL *et al*: **Obligate biotrophy features unraveled by the genomic analysis of rust fungi**. *Proceedings of the National Academy of Sciences of the United States of America* 2011, **108**(22):9166-9171.
2. Toome M, Ohm RA, Riley RW, James TY, Lazarus KL, Henrissat B, Albu S, Boyd A, Chow J, Clum A *et al*: **Genome sequencing provides insight into the reproductive biology, nutritional mode and ploidy of the fern pathogen Mixia osmundae**. *The New phytologist* 2014, **202**(2):554-564.
3. Gioti A, Nystedt B, Li W, Xu J, Andersson A, Averette AF, Munch K, Wang X, Kappauf C, Kingsbury JM *et al*: **Genomic insights into the atopic eczema-associated skin commensal yeast Malassezia sympodialis**. *mBio* 2013, **4**(1):e00572-00512.
4. Morita T, Koike H, Hagiwara H, Ito E, Machida M, Sato S, Habe H, Kitamoto D: **Genome and transcriptome analysis of the basidiomycetous yeast Pseudozyma antarctica producing extracellular glycolipids, mannosylerythritol lipids**. *PloS one* 2014, **9**(2):e86490.
5. Konishi M, Hatada Y, Horiuchi J: **Draft Genome Sequence of the Basidiomycetous Yeast-Like Fungus Pseudozyma hubeiensis SY62, Which Produces an Abundant Amount of the Biosurfactant Mannosylerythritol Lipids**. *Genome announcements* 2013, **1**(4).
6. Kämper J, Kahmann R, Bolker M, Ma LJ, Brefort T, Saville BJ, Banuett F, Kronstad JW, Gold SE, Muller O *et al*: **Insights from the genome of the biotrophic fungal plant pathogen Ustilago maydis**. *Nature* 2006, **444**(7115):97-101.
7. Laurie JD, Ali S, Linning R, Mannhaupt G, Wong P, Guldener U, Munsterkotter M, Moore R, Kahmann R, Bakkeren G *et al*: **Genome comparison of barley and maize smut fungi reveals targeted loss of RNA silencing components and species-specific presence of transposable elements**. *The Plant cell* 2012, **24**(5):1733-1745.
8. Xu J, Saunders CW, Hu P, Grant RA, Boekhout T, Kuramae EE, Kronstad JW, Deangelis YM, Reeder NL, Johnstone KR *et al*: **Dandruff-associated Malassezia genomes reveal convergent and divergent virulence traits shared with plant and human fungal pathogens**. *Proceedings of the National Academy of Sciences of the United States of America* 2007, **104**(47):18730-18735.

9. Schirawski J, Mannhaupt G, Munch K, Brefort T, Schipper K, Doehlemann G, Di Stasio M, Rossel N, Mendoza-Mendoza A, Pester D *et al*: **Pathogenicity determinants in smut fungi revealed by genome comparison**. *Science* 2010, **330**(6010):1546-1548.
10. Sharma R, Mishra B, Runge F, Thines M: **Gene loss rather than gene gain is associated with a host jump from monocots to dicots in the smut fungus Melanopsichium pennsylvanicum**. *Genome biology and evolution* 2014.
11. Floudas D, Binder M, Riley R, Barry K, Blanchette RA, Henrissat B, Martinez AT, Otillar R, Spatafora JW, Yadav JS *et al*: **The Paleozoic origin of enzymatic lignin decomposition reconstructed from 31 fungal genomes**. *Science* 2012, **336**(6089):1715-1719.
12. Janbon G, Ormerod KL, Paulet D, Byrnes EJ, 3rd, Yadav V, Chatterjee G, Mullapudi N, Hon CC, Billmyre RB, Brunel F *et al*: **Analysis of the genome and transcriptome of Cryptococcus neoformans var. grubii reveals complex RNA expression and microevolution leading to virulence attenuation**. *PLoS genetics* 2014, **10**(4):e1004261.
13. Olson A, Aerts A, Asiegbu F, Belbahri L, Bouzid O, Broberg A, Canback B, Coutinho PM, Cullen D, Dalman K *et al*: **Insight into trade-off between wood decay and parasitism from the genome of a fungal forest pathogen**. *The New phytologist* 2012, **194**(4):1001-1013.
14. Martin F, Aerts A, Ahren D, Brun A, Danchin EG, Duchaussoy F, Gibon J, Kohler A, Lindquist E, Pereda V *et al*: **The genome of Laccaria bicolor provides insights into mycorrhizal symbiosis**. *Nature* 2008, **452**(7183):88-92.
15. Collins C, Keane TM, Turner DJ, O'Keeffe G, Fitzpatrick DA, Doyle S: **Genomic and proteomic dissection of the ubiquitous plant pathogen, Armillaria mellea: toward a new infection model system**. *Journal of proteome research* 2013, **12**(6):2552-2570.
16. Stajich JE, Wilke SK, Ahren D, Au CH, Birren BW, Borodovsky M, Burns C, Canback B, Casselton LA, Cheng CK *et al*: **Insights into evolution of multicellular fungi from the assembled chromosomes of the mushroom Coprinopsis cinerea (Coprinus cinereus)**. *Proceedings of the National Academy of Sciences of the United States of America* 2010, **107**(26):11889-11894.
17. Martinez D, Larrondo LF, Putnam N, Gelpke MD, Huang K, Chapman J, Helfenbein KG, Ramaiya P, Detter JC, Larimer F *et al*: **Genome sequence of the lignocellulose degrading fungus Phanerochaete chrysosporium strain RP78**. *Nature biotechnology* 2004, **22**(6):695-700.
18. Ohm RA, de Jong JF, Lugones LG, Aerts A, Kothe E, Stajich JE, de Vries RP, Record E, Levasseur A, Baker SE *et al*: **Genome sequence of the model mushroom Schizophyllum commune**. *Nature biotechnology* 2010, **28**(9):957-963.
19. Padamsee M, Kumar TK, Riley R, Binder M, Boyd A, Calvo AM, Furukawa K, Hesse C, Hohmann S, James TY *et al*: **The genome of the xerotolerant mold Wallemia sebi reveals adaptations to osmotic stress and suggests cryptic sexual reproduction**. *Fungal genetics and biology : FG & B* 2012, **49**(3):217-226.
20. Zajc J, Liu Y, Dai W, Yang Z, Hu J, Gostincar C, Gunde-Cimerman N: **Genome and transcriptome sequencing of the halophilic fungus Wallemia ichthyophaga: haloadaptations present and absent**. *BMC genomics* 2013, **14**:617.
21. Nierman WC, Pain A, Anderson MJ, Wortman JR, Kim HS, Arroyo J, Berriman M, Abe K, Archer DB, Bermejo C *et al*: **Genomic sequence of the pathogenic and allergenic filamentous fungus Aspergillus fumigatus**. *Nature* 2005, **438**(7071):1151-1156.
22. Goffeau A, Barrell BG, Bussey H, Davis RW, Dujon B, Feldmann H, Galibert F, Hoheisel JD, Jacq C, Johnston M *et al*: **Life with 6000 genes**. *Science* 1996, **274**(5287):546, 563-547.
23. Wood V, Gwilliam R, Rajandream MA, Lyne M, Lyne R, Stewart A, Sgouros J, Peat N, Hayles J, Baker S *et al*: **The genome sequence of Schizosaccharomyces pombe**. *Nature* 2002, **415**(6874):871-880.
24. Hane JK, Lowe RG, Solomon PS, Tan KC, Schoch CL, Spatafora JW, Crous PW, Kodira C, Birren BW, Galagan JE *et al*: **Dothideomycete plant interactions illuminated by genome sequencing and EST analysis of the wheat pathogen Stagonospora nodorum**. *The Plant cell* 2007, **19**(11):3347-3368.
25. Martinez D, Berka RM, Henrissat B, Saloheimo M, Arvas M, Baker SE, Chapman J, Chertkov O, Coutinho PM, Cullen D *et al*: **Genome sequencing and analysis of the biomass-degrading**

fungus Trichoderma reesei (syn. Hypocrea jecorina). *Nature biotechnology* 2008, **26**(5):553-560.

26. Dujon B, Sherman D, Fischer G, Durrens P, Casaregola S, Lafontaine I, De Montigny J, Marck C, Neuveglise C, Talla E *et al*: **Genome evolution in yeasts**. *Nature* 2004, **430**(6995):35-44.
27. Galagan JE, Calvo SE, Cuomo C, Ma LJ, Wortman JR, Batzoglou S, Lee SI, Basturkmen M, Spevak CC, Clutterbuck J *et al*: **Sequencing of Aspergillus nidulans and comparative analysis with A. fumigatus and A. oryzae**. *Nature* 2005, **438**(7071):1105-1115.
28. Jones T, Federspiel NA, Chibana H, Dungan J, Kalman S, Magee BB, Newport G, Thorstenson YR, Agabian N, Magee PT *et al*: **The diploid genome sequence of Candida albicans**. *Proceedings of the National Academy of Sciences of the United States of America* 2004, **101**(19):7329-7334.
29. Pel HJ, de Winde JH, Archer DB, Dyer PS, Hofmann G, Schaap PJ, Turner G, de Vries RP, Albang R, Albermann K *et al*: **Genome sequencing and analysis of the versatile cell factory Aspergillus niger CBS 513.88**. *Nature biotechnology* 2007, **25**(2):221-231.
30. Kellis M, Birren BW, Lander ES: **Proof and evolutionary analysis of ancient genome duplication in the yeast Saccharomyces cerevisiae**. *Nature* 2004, **428**(6983):617-624.
31. Dietrich FS, Voegeli S, Brachat S, Lerch A, Gates K, Steiner S, Mohr C, Pohlmann R, Luedi P, Choi S *et al*: **The Ashbya gossypii genome as a tool for mapping the ancient Saccharomyces cerevisiae genome**. *Science* 2004, **304**(5668):304-307.
32. Dean RA, Talbot NJ, Ebbole DJ, Farman ML, Mitchell TK, Orbach MJ, Thon M, Kulkarni R, Xu JR, Pan H *et al*: **The genome sequence of the rice blast fungus Magnaporthe grisea**. *Nature* 2005, **434**(7036):980-986.
33. Galagan JE, Calvo SE, Borkovich KA, Selker EU, Read ND, Jaffe D, FitzHugh W, Ma LJ, Smirnov S, Purcell S *et al*: **The genome sequence of the filamentous fungus Neurospora crassa**. *Nature* 2003, **422**(6934):859-868.
34. Ma LJ, Ibrahim AS, Skory C, Grabherr MG, Burger G, Butler M, Elias M, Idnurm A, Lang BF, Sone T *et al*: **Genomic analysis of the basal lineage fungus Rhizopus oryzae reveals a whole-genome duplication**. *PLoS genetics* 2009, **5**(7):e1000549.
