# Supplementary material for: The genome of the basal agaricomycete Xanthophyllomyces dendrorhous provides insights into the organization of its acetyl-CoA derived pathways and the evolution of Agaricomycotina
Source: BMC Genomics. 2015 Mar 25;16(1):233. doi: 10.1186/s12864-015-1380-0 (PMC4393869; doi:10.1186/s12864-015-1380-0)
Supplement: Additional file 2: Figure S2. — Phylogenetic tree based on Bayesian phylogenetic inference. Numbers on branches denote posterior probabilities. [file 12864_2015_1380_MOESM2_ESM.pdf]

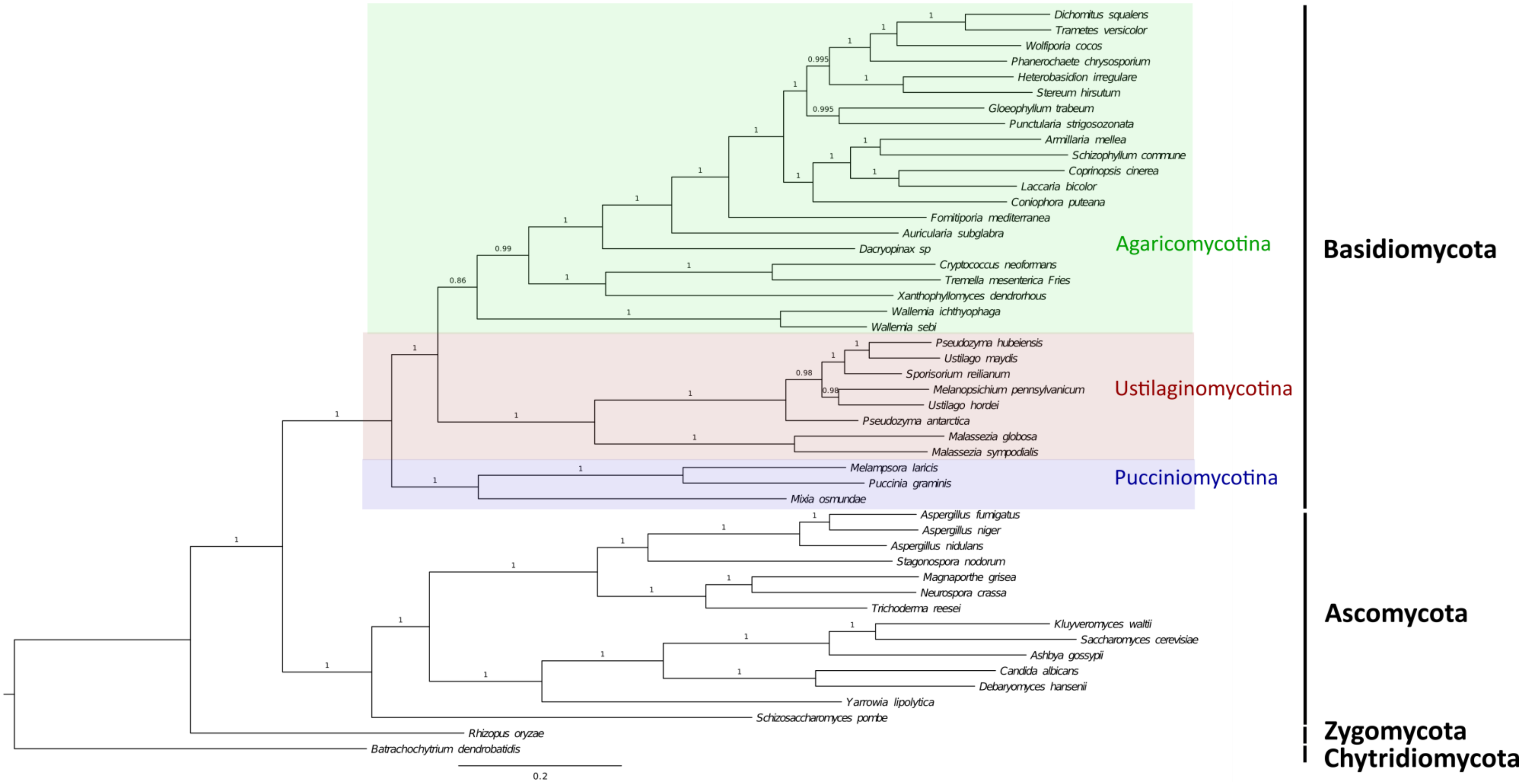

Supplementary Figure 2. Phylogenetic tree based on Bayesian phylogenetic inference. Numbers on branches denote posterior probabilities.
